# Epidemiological and Clinical-Laboratorial Profile of Schistosomiasis in an Endemic Urban Area in Pernambuco, Brazil

**DOI:** 10.1007/s11686-026-01384-0

**Published:** 2026-09-07

**Authors:** Maria Eduarda da Silva Pereira, Walter Lins Barbosa Júnior, Diego Leandro Reis da Silva Fernandes, Ana Lúcia Coutinho Domingues, Virginia Maria Barros de Lorena, Elainne Christine de Souza Gomes

**Affiliations:** 1 Center of Medical Sciences, Federal University of Pernambuco, Recife, PE Brazil; 2 Immunology Department, Fundação Oswaldo Cruz, Instituto Aggeu Magalhães, Recife, PE Brazil; 3 Instituto Nacional de Pesquisa do Pantanal, Ministry of Science, Technology and Innovation, Cuiabá, MT Brazil; 4 Parasitology Department, Fundação Oswaldo Cruz, Instituto Aggeu Magalhães, Recife, PE Brazil; 5 Gastroenterology Department, Hospital das Clínicas/EBSERH, Federal University of Pernambuco, Recife, Brazil

**Keywords:** Schistosomiasis mansoni, Urban transmission, Epidemiology, Fibrosis

## Abstract

In Brazil, although schistosomiasis mansoni is estimated to present positivity and severe infection rates below 5% in endemic states, there are inequalities among regions represented by municipalities showing moderate and high positivity rates. This study aimed to describe the epidemiological and clinical-laboratory profiles of an urban area. A cross-sectional ecological study was conducted in Novo Horizonte, Jaboatão dos Guararapes, state of Pernambuco, Brazil. The study area was mapped using a GPS receiver, a parasitological survey was performed (using Kato-Katz and Hoffman, Pons e Janer techniques), and clinical evaluations were conducted using upper abdominal ultrasonography (US) and socioenvironmental conditions related to household and peridomestic environments were performed. Based on parasitological techniques, 23.68% positivity rate was found, 66.67% male individuals, and a 284.5 eggs per gram mean. Using molecular biology techniques, the prevalence has increased to 28.94%. US was performed in 55 infected and 57 non-infected individuals, of which 31 and 19 presented periportal fibrosis (PPF), respectively. Infected individuals with PPF presented a larger portal vein diameter and had a higher frequency in males. The Novo Horizonte showed a moderate positivity rate within a municipality considered to have low endemicity. The results highlight the need to carry out frequent parasitological surveys, increased active case finding and the use of other more sensitive diagnosis methods. More targeted studies are needed to better understand the dynamics of schistosomiasis transmission in this locality, as well as the health consequences for exposed and infected individuals.

## Introduction

Schistosomiasis is a neglected tropical disease (NTD) caused by trematodes of the genus *Schistosoma*, affecting approximately 250 people across 74 countries, mainly in tropical and subtropical regions [1]. It is estimated to account for approximately 1.7 million disability-adjusted life years (DALYs) worldwide, highlighting its significant impact on affected populations [2]. The main *Schistosoma* species causing the disease are *S. mansoni*, *S. japonicum*, *S. haematobium*, *S. intercalatum*, and *S. mekongi*. In Brazil, *S. mansoni* is responsible for the disease [3].

Clinical manifestations of *Schistosoma manson*i infection can be categorized into acute and chronic phases. The acute phase may present cercarial dermatitis, diarrhea, fever, abdominal pain, and eosinophilia. Those clinical manifestations are more common among individuals travelling to endemic areas or during primary infection, being uncommon in individuals living in those areas [4]. Chronic manifestations result from the granulomatous inflammatory response to parasite eggs in the liver and subsequent development of periportal fibrosis (PPF) [5], which can cause blockage of the main portal vein, leading to clinical manifestations that vary in severity, such as hepatosplenomegaly, portal hypertension, esophageal varices, and in some cases upper gastrointestinal bleeding, which may be fatal [6, 7].

In Brazil, schistosomiasis is estimated to affect around 2.6% of the population [8], with a percentage of positivity and severe infections below 5% in endemic states; however, there are municipalities presenting moderate and high positivity rates [8], indicating inequality among regions. Inequalities are also observed in hospitalization rates across the country, with Pernambuco having the highest hospitalization rate among states in the period of 2014 to 2019 [8] and the highest proportion of deaths among the states in the Northeast region of Brazil [9], even though it is not the state with the highest number of positive cases [10].

Another important epidemiological aspect is related to the urbanization of schistosomiasis. In Brazil and worldwide, transmission has been associated to rural areas; however, urban expansion and rural exodus, combined with lack of sanitation, recurrent flooding, and the presence of snail intermediate hosts, have facilitated the emergence and maintenance of urban transmission foci [11, 12]. This scenario has become common in the state of Pernambuco, characterizing new endemic areas with a profile of accidental exposure. In these areas, individuals are exposed to a daily risk of infection when traversing flooded streets during periods of heavy rainfall and urban flooding [13, 14, 15].

This scenario highlights the importance of understanding the epidemiological and clinical profiles across different endemic municipalities in the country. Therefore, the aim of this study was to describe the epidemiological and clinical-laboratory profiles of an area within the endemic municipality of Jaboatão dos Guararapes, located in the Recife Metropolitan Region (RMR), Pernambuco.

## Materials and Methods

### Study Location

The study was conducted in the endemic locality of Novo Horizonte, in Barra de Jangada, within the municipality of Jaboatão dos Guararapes, Pernambuco, Brazil, between April and June 2024. The study site was selected based on epidemiological data obtained from the Schistosomiasis Control Program Information System (SISPCE), as it is recognized as an endemic area for the disease. Jaboatão dos Guararapes is part of the Metropolitan Region of Recife, located approximately 17 km from the capital and has a territorial area of approximately 258.8 km^2^. According to the most recent census conducted by the Brazilian Institute of Geography and Statistics (IBGE) in 2022, the municipality has a population of 644,037 inhabitants, considered the second most populous municipality in the state of Pernambuco. The population density is 2489.28 inhabitants per km^2^. According to the IBGE data from 2021, Barra de Jangada has a population of 39,962 inhabitants and according to the Citizen’s Electronic Health Record (Ministry of Health), Novo Horizonte locality has a population of 4720 individuals.

### Study Design

This was a cross-sectional ecological study. The study population consisted of residents of the Novo Horizonte area who agreed to participate. The study area was mapped on the ground using Global Positioning System (GPS) technology and a census-based parasitological survey was conducted, and clinical assessments of the participants and socioenvironmental conditions related to their domestic and peridomestic environments were performed.

Sample size was calculated using Epi Info 7.2.7.0 (CDC, Atlanta, EUA) through StatCalc - Sample Size and Power mode considering a total population of 4720 residents of Novo Horizonte. Parameters were based on an estimated prevalence of 15.7% [16], 95% Confidence Interval (IC), acceptable error of 5% error and Design Effect of 1,0 and two clusters. After adjusting to a refusal/loss rate of 20%, the calculated minimum sample size was 236 individuals.

### Locality Mapping

The locality was georeferenced using Garmin Montana 650 (Garmin International, Olathe, KS) handheld GPS technology with a Universal Transverse Mercator (UTM) projection Datum SIRGAS 2000 configured as a receiver. The location was mapped on the ground by walking and we considered the block as the unit of mapping. Subsequently, GPS data was transferred and stored as shapefiles. All households with at least one study participant were georeferenced. The software used for spatial distribution and map creation was QGIS 3.16.16. Street and shapefiles were extracted from IBGE Block-Face Database for Brazilian Addresses organized by state (UF)/municipalities and Territorial Meshes, respectively [17]. Vector data for waterbodies were extracted from the primary source OpenStreetMap (OSM) using the QGIS QuickOSM tool. Satellite imagery was obtained via Google Earth Pro Desktop and georeferenced in QGIS using the GDAL Georeferencer plug-in.

### Parasitological Survey

A census-based parasitological survey was conducted using coproscopic tests on the population living in two microareas covered by the Family Health Care Unit of Barra de Jangada I in Novo Horizonte. All residents of the study area were invited and voluntarily enrolled to participate. Participants did not receive any compensation for participating in the surveys. After providing written informed consent, participants received a plastic container to collect a stool sample, identified with the participant’s name and household identification number.

Samples were collected and transported to the laboratory for analysis using the Kato-Katz (KK) method, performed with Helm Test kit (Biomanguinhos, Fiocruz, Rio de Janeiro, Brazil), using two slides fecal sample, which identifies schistosomiasis cases by finding parasite eggs [18]. This method also allows parasite quantification of parasite burden (number of eggs per gram (EPG) of stool). Cases were classified by the intensity of infection, according to World Health Organization (WHO) criteria: 1–99 EPG (light infection), 100–399 EPG (moderate infection), and > 400 EPG (heavy infection) [19].

Part of the samples from individuals KK negatives were further analyzed using Hoffman, Pons e Janer (HPJ) parasitological technique, followed by real-time polymerase chain reaction (qPCR). First, DNA was extracted using the QIAamp Fast DNA Stool Mini Kit (Qiagen, Hilden, Germany) following manufacturer instructions. DNA was stored at − 20 °C after extraction. Next, qPCR was performed for the detection of parasite DNA [20, 21]. TaqMan Reagents Exogenous Internal Positive Control (IPC) was used to evaluate all samples and distinguish negative samples from the presence of inhibitors. Ultrapure walter was used as a No Template Control (NTC). 10X Block-Exp IPC was used as the No Amplification Control. DNA extracted from adult male worms was used a positive control.

Reaction was performed using a final volume of 22 µL containing 10 µL of TaqMan Universal PCR Master Mix, 2 µL of Forward and Reverse primer each (10 pmol/µL), 2 µL of PO2 probe (10 pmol/µL), 4 µL of ultrapure water and 2 µL of extracted DNA. The second reaction was performed using a final volume of 21 µL containing 10 µL of TaqMan Universal PCR Master Mix, 5 µL of 10X Exogenous IPC Mix, 1 µL of 50X Exogenous IPC Mix, 3 µL of ultrapure water and 2 µL of extracted DNA. Threshold cycles (Ct) lower than 30.737 were considered as a positive result. QuantStudio 5 Real-Time PCR System (Applied Biosystems, Thermo Fisher Scientific, Waltham, MA, USA) was used for all reactions.

The objective was to obtain a group of truly negative individuals that were used to compare the results related to clinical and socioenvironmental assessment. For this purpose, we performed the analysis (HPJ and qPCR) on a sample size twice as large as the number of Kato-Katz positive individuals, allowing the formation of two groups of similar size after excluding individuals. Therefore, the positive group included positives from one of the two parasitological tests (KK and HPJ), and the negative group included those negatives for all diagnostic methods (KK, HPJ and qPCR).

The prevalence of schistosomiasis in the locality was calculated by the ratio of the total number of parasitological positive individuals per total number of examined individuals multiplied by 100.

At the end of the analysis, all the individuals that were enrolled on parasitological survey received their test results, and the list of all positive cases were sent for treatment with praziquantel in accordance with the Brazilian Schistosomiasis Surveillance and Control Program guidelines.

### Clinical Examination by Ultrasonography (US)

All individuals who tested positive for *S. mansoni* by KK or HPJ parasitological techniques and individuals who tested negative by all three techniques, were invited for clinical evaluation using upper abdominal ultrasonography. On the same day, a questionnaire for socioenvironmental data collected was applied for each participant.

During the examination, liver assessment included the presence or absence of hepatic steatosis (classified as mild, mild-moderate, moderate or severe), chronic liver disease (CLD) and/or periportal fibrosis. Periportal fibrosis was classified according to the Niamey protocol [22] as follows: absence of PPF (pattern A), doubtful PPF (pattern B - described as diffuse echogenic foci), peripheral fibrosis (pattern C - described as highly echogenic “ring echoes” as “pipe stems”), central fibrosis (pattern D - described as highly echogenic “ruff” around portal bifurcation and main stem), peripheral and central fibrosis (pattern Dc - combined D and C patterns), advanced fibrosis (pattern E - described as highly echogenic “patches” that extends from the pain portal vein and branches into the parenchyma) and very advanced fibrosis (pattern F - described as highly echogenic “bands” and “streaks” that extends from the vain portal vein and its bifurcation to the liver surface causing liver retraction). Measurements included the sizes of the left and right liver lobes, splenic index (spleen volume was calculated based on longitudinal and transversal measurements), and portal vein diameter. The presence of ascites and collateral circulation was also recorded when observed.

All ultrasound examinations were performed by the same blind-examiner, a member of the research group, who is specialized and experienced in imaging diagnosis, especially in the context of schistosomiasis and who is part of the specialized outpatient clinic for Schistosomiasis at a referral hospital of the Federal University of Pernambuco. All examinations were performed using a portable LOGIQ *e* ultrasound with a 4.5 MHz 4 C-RS convex transducer (GE Health Care, Chicago, Illinois, United States).

### Socioenvironmental Data Collection

The socioenvironmental data collected included duration of residence in the area, education level, occupation, previous exposure to freshwater, time since last exposure, and type of exposure, street pavement conditions, presence of open sewage, rainwater accumulation, exposure to flooding, seasonality of exposure, schistosomiasis and treatment previous history, other parasitic infections and treatment previous history, medical history (blood transfusion, splenectomy, ascites, blood vomiting, jaundice, arterial hypertension, diabetes, hepatitis and HIV infection) and alcohol consumption.

### Statistical Analysis

All data was collected using a Google Sheet (Google LLC) and analyzed using Graph Pad Prism 8.0.2 software (GraphPad Software, San Diego, California USA). Shapiro-Wilk test applied to evaluate date normality (normal distribution was considered when a *p-value* < 0.05). Descriptive statistics were used to report data on age group, sex, parasite load, clinical (left and right liver lobes sizes, splenic index and portal vein diameter) and socioenvironmental data.

Based on quantitative data distribution (age, parasite load and clinical data), statistical analysis was performed using unpaired* t* test was used for parametric data and Mann-Whitney test for non-parametric data when comparing two groups. Ordinary one-way ANOVA (followed by Tukey multiple comparisons post-hoc test) was used for parametric data and Kruskal-Wallis test (followed by Dunn post-hoc test) for non-parametric data when comparing more than two groups. For categorical data (socioenvironmental data and sex), qui-square test was used. A *p-value* of < 0.05 was considered as statistically significant for all analysis performed.

### Ethics Statement

All participants, or their legal guardians, from whom information and biologic samples were obtained, or clinical procedures were performed, provided written informed consent by signing the Free and Informed Consent and/or Assent Form. The study was approved by the Ethics and Research Committee from the Federal University of Pernambuco, Recife, Brazil (CAAE 0022.0.107.000–08), under number 5.905.584.

## Results

### Parasitological Survey Results

**Fig. 1:**
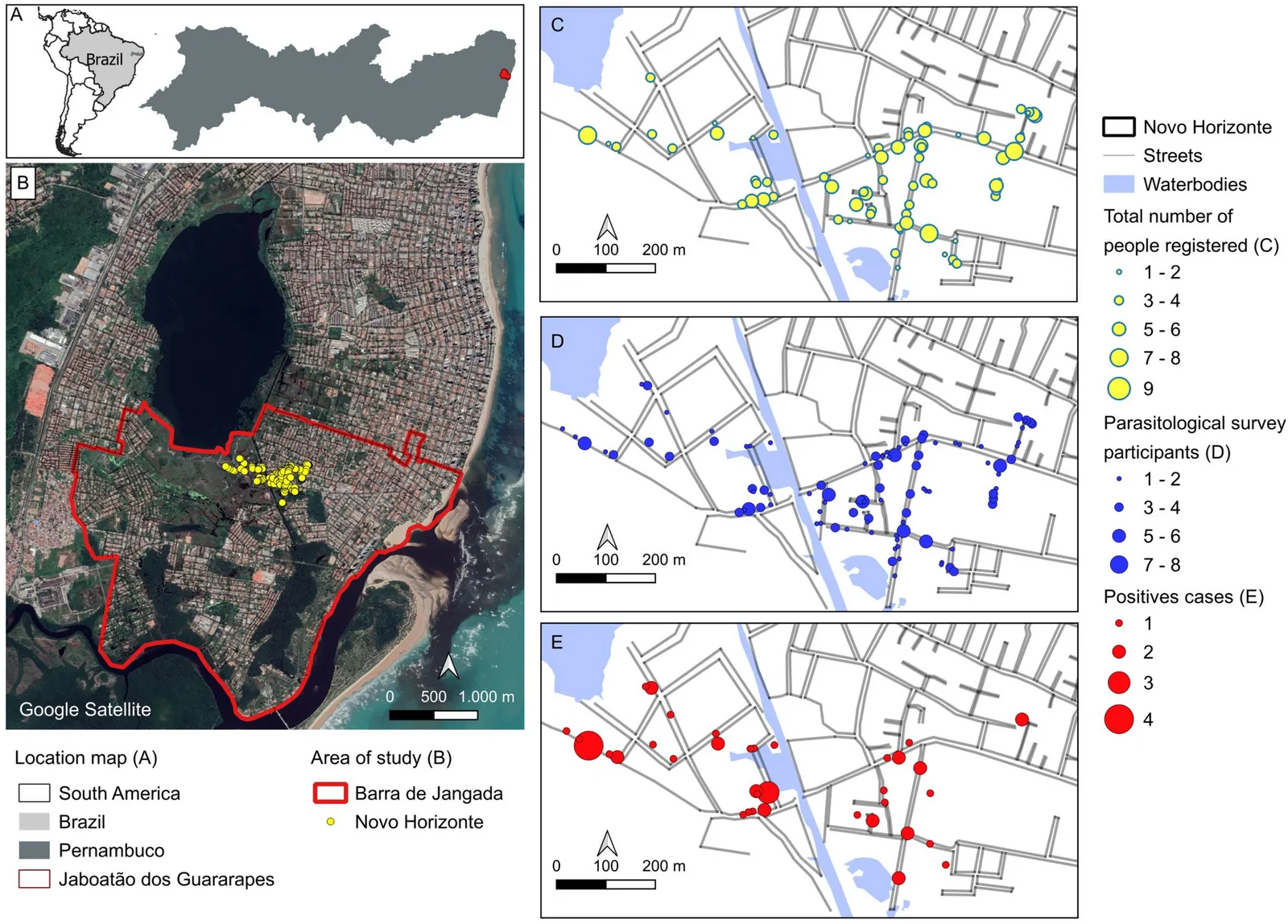
Study area and the distribution of registered individuals, sampled and positive cases in Novo Horizonte, Barra de Jangada, Jaboatão dos Guararapes - Pernambuco, 2024

A total of 416 residents were registered during the survey (Fig. 1A–C). Of these individuals, 52.40% were male (218/416) and 47.60% (198/416) female, with a mean age of 31.60 years (0–88 years; SD ± 20.98). A total of 266 stool samples (63.94%) were obtained from the registered individuals and analyzed by the Kato-Katz method. From those participants, 52.26% (139/266) were male, with a mean age of 34.94 years (± 21.22) and median age of 36 (1–86) years; SD ± 21.22). From the 150 (36.06%) registered non-participants individuals who did not provide a stool sample, 52.67% (79/150) were male (*p* = 0.9358) with a mean age of 25.38 (± 25.38) years (*p* < 0.0001) and median age of 21.50 (0–88) years (*p* < 0.0001).

From Fig. 1D, it is also possible to see that parasitological survey participants are geographically represented as the total number of registered people invited to be part of research (Fig. 1C). Analyzing the positive distribution of cases (Fig. 1E) it is noticed that cases occur all over the study area and that the house with the highest number of cases (4 cases) is located closest to the water collection.

According to Kato-Katz two-slides analysis, 78.57% of the samples were negative (209/266) and 21.43% were positive for *S. mansoni* infection (57/266), with one *Ascaris lumbricoides* co-infection. Regarding other helminths, among the *S. mansoni* negatives, one individual had a positive result for *Trichuris trichiura* and another for *A. lumbricoides*. The infection intensity analysis showed that almost 50% of the cases present light parasite load (Table 1).

HPJ techniques were performed on 99 of the 209 KK negative samples, identifying 6 more *S. mansoni* positives, totaling 63 positive individuals, of which 66.67% (42/63) were male (*p* = 0.0088) with a mean age of 38.08 years (6–81 years; SD ± 19.28) (Table 1).

**Table 1:** Kato-Katz, Hoffman, Pons e Janer and both techniques combined analysis from one stool sample - Novo Horizonte, Jaboatão dos Guararapes, Pernambuco, 2024

	S. mansoni positives	S. mansoni negatives	Total	*p* value
KK and HPJ analysis result	63 (23.68%)	203 (76.32%)	266	
Male	42 (66.67%)	97 (47.78%)	139	**0.0088** ^a^
Female	21 (33.33%)	106 (52.22%)	127	
Age (mean) ± SD	37.89 (6–80) ± 19.26	34.01 (1–86) ± 21.76		
Age (median)	37.60 ± 19.27	34.01 ± 21.73		0.2210^b^
Eggs per gram mean (EPG) ± SD	37.5 (6–80)	34 (1–86)		0.2172^c^
Eggs per gram median (EPG)	96 (24−3168)	–		
Light parasite load	30 (47.62%)	–		
Moderate parasite load	14 (22.22%)	–		
Heavy parasite load	13 (20.64%)	–		
No information	6 (9.52%)	–		
Other parasites				
*Ascaris lumbricoides*	0	1		
*Trichuris trichiura*	1	1		
Ancylostomatidae	0	0		

a: Chi-square test

b: Unpaired* t* test

c: Mann-Whitney test

Bold: p < 0.05 was considered

Samples that had negative results for both techniques and had sufficient material for DNA extraction were subjected to DNA extraction protocol followed by amplification using the qPCR technique. 85 samples were analyzed by molecular biology, of which 16.47% (14/85) had positive qPCR amplification results and were excluded from recruitment for clinical evaluation and socioenvironment survey. Thus, using the diagnostic techniques, 63 individuals were identified as positive for *S. mansoni* and 71 negatives. All these individuals were recruited for clinical examinations.

### Socioenvironmental Survey Results

According to the socioenvironmental survey, among the positive individuals identified through the epidemiological survey, none had lived in the endemic area for less than 1 year and 80% reported having lived there for at least five years. Regarding education, 40% of the population had only incomplete elementary education, 18.18% had incomplete secondary education, 23.63% had completed high school, and only 5.54% had higher education. Results were similar among negative individuals, where 43.85% had incomplete elementary education, 12.28% had incomplete secondary education, 29.82% had completed high school, and only 1.75% had higher education.

Approximately 69% of positive individuals reported having had previous contact with freshwater, 42.10% of those less than a year ago. Similarly to negative individuals, 70.18% reported previous contact with freshwater, 22.50% of those less than a year ago.

Regarding sanitation conditions, only 10.90% and 8.77% of the positive and negative individuals, respectively, lived on paved streets; 74.54% and 70.18% of the positive and negative individuals, respectively, reported open septic tanks leaking on the street where they lived.

Similarly, around 98% of both groups reported water accumulation on the street, of which only two positives and three negatives reported having means of getting around flooded areas without exposure, thus avoiding and not being forced to come into contact with water.

Four infected (7.27%) and four negative individuals (7.02%) reported a previous diagnosis of schistosomiasis. However, seven positives (12.72%) and six (10.53%) negatives reported previous treatment, all positives more than five years ago. No individual reported a history of hepatitis, one individual reported diabetes mellitus and regarding alcohol consumption, 38.18% positive and 12.28% negative individuals reported some level of consumption (Table 2).

**Table 2:** Socioenvironmental survey applied for negative and positive individuals from Novo Horizonte, Jaboatão dos Guararapes, Pernambuco, 2024

	S. mansoni positives	S. mansoni negatives	*p* value
Individuals	55	57	
*Time spent living in the location*			
< 1 year	0 (0%)	6 (10.52%)	
1–4 years	9 (16.36%)	5 (8.78%)	0.0081^a^
5 years or more	44 (80%)	45 (78.95%)	0.0187^a^
Missing information	2 (3.64%)	1 (1.75%)	
*Level of education*			
None	5 (9.10%)	4 (7.02%)	
Incomplete primary education	22 (40%)	25 (43.86%)	0.6304^a^
Complete primary education	11 (20%)	7 (12.28%)	0.7818^a^
Complete secondary education	14 (25.45%)	17 (29.82%)	0.5825^a^
Bachelor’s degree/post-graduation	3 (5.45%)	1 (1.75%)	0.5060^a^
Missing information	0 (0%)	3 (5.26)	
*Freshwater previous contact*			
No	17 (30.90%)	17 (29.82%)	
Yes	38 (69.10%)	40 (70.18%)	0.9007^a^
*Paved street*			
No	49 (89.09%)	52 (91.23%)	
Yes	6 (10.90%)	5 (8.77%)	0.7040^a^
*Open sewage*			
No	14 (25.46%)	17 (29.82%)	
Yes	41 (74.54%)	40 (70.18%)	0.6053^a^
*Rainwater accumulation*			
No	1 (1.82%)	1 (1.75%)	
Yes	54 (98.18%)	56 (98.25%)	0.9797^a^
*Exposure to floods*			
No	2 (3.64%)	3 (5.26%)	
Yes	53 (96.36%)	54 (94.74%)	0.6769^a^
*Previous schistosomiasis history*			
No	51 (92.73%)	53 (92.98%)	
Yes	4 (7.27%)	4 (7.02%)	0.9582^a^
*Previous schistosomiasis treatment*			
No	48 (87.28%)	51 (89.47%)	
Yes	7 (12.72%)	6 (10.53%)	0.7162^a^
< 5 years	0 (%)	4 (66.67%)	
> 5 years	7 (100%)	2 (33.33%)	0.0094^a^
*Hepatitis*			
No	55 (100%)	55 (96.49%)	
Yes	0 (0%)	2 (3.51%)	0.1610^a^
*Diabetes*			
No	54 (98.19%)	51 (89.47%)	
Yes	1 (1.81%)	6 (10.53%)	0.0570^a^
*Alcohol consumption*			
None	34 (61.82%)	50 (87.72%)	
Some	21 (38.18%)	7 (12.28%)	0.0016^a^

a: Chi-square test

### Clinical Variables According to Ultrasonography Examination Results

All 63 infected and 71 non-infected individuals were recruited for upper abdomen ultrasonography examination, to which 55 and 57 attended, respectively. Among positives, 14 had no fibrosis (pattern A or B), 50% of whom were male, with a mean age of 21 years (5–58 years; SD ± 13.80) and a mean parasite load of 161.50 EPG (24–552 EPG); 31 individuals had fibrosis C, D/Dc, E or F, 74.19% (23/31) of whom were male, with a mean age of 41.10 years (7–80) and a mean parasite load of 330.4 EPG (24− 3168 EPG); and 10 had liver steatosis or chronic liver disease (Table 3).

Among infected individuals, the no fibrosis group had a mean age of 21 years (SD ± 13.88 years), which was significantly lower than the group with fibrosis, which had a mean age of 41.10 years (SD ± 18.26 years), and the group with steatosis or chronic liver disease, which had a mean age of 45 years (SD ± 17.24 years) (*p* = 0.0009). This result showed a similar pattern when comparing the means of the group of uninfected individuals (*p* < 0.0001) and median ages also in the infected group (*p* = 0.0012) and uninfected individuals (*p* = 0.0004). Furthermore, among those who tested positive, the fibrosis group presented a larger portal vein diameter than the group without fibrosis (*p* = 0.0459). Among the negative results, only the group with steatosis or chronic liver disease showed a statistically significant difference compared to the group without fibrosis (*p* = 0.1030).

**Table 3:** Sociodemographic and clinical description of individuals who had upper abdomen examinations by ultrasonography

	Variables	No fibrosis	Fibrosis	Hepatic steatosis or CLD	*p* value
Positives *n* = *55*	Individuals	14	31	10	
	Age (mean) ± SD (years)	21.00 ± 13.88	41.10 ± 18.26	45.00 ± 17.24	**0.0009** ^**a**^
	Range	5–54	7–80	17–73	
	Age (median)	20	42	51	**0.0012** ^**b**^
	Male	7 (50%)	23 (74.19%)	5 (50%)	0.1807^c^
	Female	7 (50%)	8 (25.81%)	5 (50%)	
	Parasite load mean (EPG)	161**.**5 ± 171.2	330.4 ± 656.6	483.6 ± 534.5	0.3784^d^
	Parasite load median (EPG) (min–max)	96 (24—552)	84 (24 – 3,168)	312 (24–1,800)	0.1114^e^
	Spleen index mean ± SD (cm)	37.40 ± 12.02	39.72 ± 21.98	53.15 ± 33.45	0.2343^f^
	Left liver lobe size mean ± SD (cm)	7.92 ± 1.04	7.96 ± 1.46	8.27 ± 1.81	0.8049^ g^
	Right liver lobe size mean ± SD (cm)	13.02 ± 2.04	13.43 ± 1.52	14.74 ± 1.77	0.0519^ h^
	Portal vein diameter mean ± SD (cm)	0.88 ± 0.19	1.02 ± 0.19	0.93 ± 0.181	**0.0459** ^**i**^
Negatives *n* = *57*	Individuals	29	19	9	
	Range	2–62	10–85	30–76	
	Age (mean) ± SD (years)	23.86 ± 17.11	44.11 ± 20.12	49.78 ± 12.89	**<0.0001** ^**j**^
	Age (median)	19	49	48	**0.0004** ^** k**^
	Male	16 (55.17%)	9 (47.37%)	6 (66.67%)	0.1807^ l^
	Female	13 (44.83%)	10 (52.63%)	3 (33.33%)	
	Splenic index ± SD (cm^2^)	31.22 ± 11.90	37.75 ± 14.03	37.98 ± 14.39	0.1260^ m^
	Left liver lobe size mean ± SD (cm)	7.0**6** ± 1.16	8.0**2** ± 1.44	7.**90** ± 1.2**5**	0.0295^n^
	Right liver lobe size mean ± SD (cm)	12.52 ± 2.14	13.52 ± 1.**40**	15.31 ± 2.67	0.0026^o^
	Portal vein diameter mean ± SD (cm)	0.84 ± 0.1**7**	0.92 ± 0.15	1.060 ± 0.1**4**	**0.0103** ^**p**^

CLD: Chronic liver disease

a: Kruskal-Wallis test and Tukey’s multiple comparisons test (group 1 vs. group 2: *p* = 0.0017; group 1 vs. group 3: *p* = 0.0037)

b: ANOVA and Dunn’s multiple comparisons test (group 1 vs. group 2: *p* = 0.0035; group 1 vs. group 3: *p* = 0.0042)

c: Chi-square test

d: ANOVA

e: Kruskal-Wallis test

f: Kruskal-Wallis test

g: Ordinary one-way ANOVA test

h: Ordinary one-way ANOVA test

i: Kruskal-Wallis test and Tukey’s multiple comparisons test (group 1 vs. group 2: *p* = 0.0495; group 1 vs. group 3 and group 2 vs. group 3: p = not significant)

j: Kruskal-Wallis test and Tukey’s multiple comparisons test (group 1 vs. group 2: *p* = 0.0008; group 1 vs. group 3: *p* = 0.0009)

k: ANOVA: 0.0012 and Dunn’s multiple comparisons test (group 1 vs. group 2: *p* = 0.0033; group 1 vs. group 3: *p* = 0.0048)

l: Chi-square test

m: Kruskal-Wallis test

n: Ordinary one-way ANOVA test (group 1 vs. group 2: *p* = 0.0354; group 1 vs. group 3: *p* = 0.2019; group 2 vs. group 3: *p* = 0.9718)

o: Ordinary one-way ANOVA test (group 1 vs. group 2: *p* = 0.2249; group 1 vs. group 3: *p* = 0.0019; group 2 vs. group 3: *p* = 0.0817)

p: Ordinary one-way ANOVA test (group 1 vs. group 2: *p* = 0.2233; group 1 vs. group 3: *p* = 0.0102; group 2 vs. group 3: *p* = 0.1600)

Bold: p < 0.05 was considered

## Discussion

During the coproscopic survey conducted in Novo Horizonte, using parasitological diagnostic techniques, a positivity rate of 23.68 was observed, with 4.88% of individuals having heavy infection intensity (≥ 400 OPG). However, when molecular techniques were applied to samples from 85 individuals with negative KK and HPJ results, an additional 14 positive cases were identified as positive, increasing the estimated prevalence to 28.94%. This rate is expected to be even higher if the test were applied to all samples obtained, due to greater sensitivity of the qPCR technique compared to parasitological techniques [20].

There has been a decrease in the number of municipalities actively participating in the Schistosomiasis Control Program (PCE) and execution of stool examinations [23] in Brazil. This fragility on performance of examinations is also observed in Pernambuco, where in 2024, 240,436 coproscopic exams were expected to be performed in schistosomiasis-endemic municipalities; however, only 42.93% of those exams were completed [24], which could underestimate prevalence in the state. When analyzing data from The State’s Health Regions III and XII, which showed the highest coverage rates, 50.83% and 60.37%, respectively, it is also possible to observe the highest positivity rates (2.72% e 2.52%, respectively) [24], suggesting that active case find could help identify cases that would not be reported otherwise.

Jaboatão dos Guararapes, included in Health Regions I, had 11,561 scheduled examinations, but only 30.23% coverage was achieved, which resulted in a positivity rate of 3.20% in 2024 [24]. This positivity rate qualifies this municipality as a low endemic area [19]; however, a low coverage and activity of the PCE campaigns may be underestimating the real epidemiological situation in the municipalities and Pernambuco as a whole [25]. This is also suggested by the identification of high endemicity areas such as Novo Horizonte in this study in a low endemicity municipality.

Due to practically and cost, Kato-Katz method remains as the recommended technique [1], especially for coproscopic surveys, however, its reduced sensitivity for individuals with low parasite burden [26] remains as an obstacle to determining prevalence and it should be taken into consideration when interpreting endemicity rates in Brazil [23]. The underestimation of schistosomiasis prevalence by parasitological methods in endemic areas also has important implications for control strategies, as active cases detection and treatment approaches depend on the local epidemiological profile. According to the Brazilian Ministry of Health guidelines, in areas with positivity rates between 15% and 25%, it is recommended to treat all positive cases and their household contacts; whereas in locations with positivity rates above 25%, it is recommended to treat all individuals and those areas should be prioritized for control actions [27]. This could also impact how actions on vigilance would be applied in Jaboatão dos Guararapes.

Regardless of the prevalence rate, active case finding in endemic areas is recommended to interrupt the transmission cycle and prevent the development of severe disease forms, thus allowing these individuals to be identified before significant hepatic damage occurs. However, active case-finding examinations are not being implemented at adequate levels. Nationally, municipalities with decline in PCE activity are also showing an increase in the number of deaths [23]. In Pernambuco, between 2015 and 2024, 1528 deaths from the disease were recorded, including 111 deaths in the last year, resulting in a mortality rate of 1.23 per 100,000 inhabitants [24]. This indicated that severe clinical forms continue to occur in the State and that early diagnosis is still insufficient. Furthermore, higher concentration of cases was observed in Health Region I, which had low coverage of exams (23.90%), occupied third place in positivity rates (2.30%) and had the lowest percentage of treatment coverage (66.40%). This is the Health Region that also includes the municipality of Jaboatão dos Guararapes [24].

Regarding the findings of this study, it was observed that the two populations of registered non-participant and participant individuals were homogeneous regarding sex. However, participants who provided stool samples had a higher median age than registered non-participants individuals who did not, this a reluctance among younger residents to provide stool samples, which is something that is observed during field work experience. Although the transmission profile in urban areas is not associated with labor activity, since all residents can be exposed to infection by walking during floods [13] and our results did not find an association between age and *S. mansoni* infection in this area, this limitation should be taken into consideration when interpreting results.

When comparing age among infected and non-infected individuals, those who had positive parasitological results had a mean age of 37.89 years, which is consistent with findings from other studies that analyzed urban areas that have reported an association between infection and ages older than 20 years [28, 29]. Positivity rates were higher in males, also corroborating data published in the literature on the epidemiological profile of urban endemic areas for schistosomiasis [16, 28, 29, 30].

Regarding the epidemiological history of the study area, in 2011 an epidemiological assessment of the schistosomiasis situation in Jaboatão dos Guararapes obtained a positivity rate of 15.7% in Novo Horizonte and 8.9% in Barra de Jangada, based on 2373 and 1220 examinations, respectively. The study also indicates that after collective treatment, positivity rates decreased by 3.5% and 2.3% in Novo Horizonte and Barra de Jangada, respectively. However, in this present study, an increase in the positivity rate was again observed after more than two decades, highlighting the maintenance of the cycle in the environment and the inability to completely interrupt the transmission cycle solely through collective treatment, particularly in settings where inadequate sanitary conditions persist [16, 28, 31] and close proximity to water bodies harboring intermediate snail hosts [32], as it is observed in Novo Horizonte. Furthermore, poor sanitation and precarious environmental conditions were evident, when observed in our study that 90.17% reported a lack of paved streets and 27.67% reporting open sewage in the streets where they live, as well as more than 95% reporting water accumulation and exposure to flooding in their residential areas. These findings evidences that measures focused only on treatment and partial improvements in sanitation only manage to reduce the positivity rate momentarily, as it tends to rise again over time, as was observed in a study that used epidemiological, environmental, and biological data to construct a mathematical model for predicting the occurrence of schistosomiasis [33], these measures are not sufficient to control or eliminate the disease.

Other social factors were analyzed and we found no statistically significant differences were observed between individuals who tested positive and those who tested negative in the area regarding education level but more than 40% of individuals had incomplete primary education in body groups. The data on education are consistent with the findings of Figueiredo et al. [34], in which most participants had completed only primary education [34]. No statistically significant difference was also found considering prior freshwater exposure and street sanitation conditions in the area, which could be explained by how residents of Novo Horizonte are mostly all exposed to the same poor sanitary conditions. Also, this area is known for its flooding history affecting most streets. We found statistically significant results when analyzing time of residence, with most positive individuals having lived in the locality for more than five years which is consistent with previous work showing association in individuals with a longer exposure time [35].

The clinical evaluation using abdominal ultrasound aimed to assess the degree of periportal fibrosis in residents, as well as spleen, left and right liver lobes measurements and portal vein diameter. It is important to highlight that among positives, ten individuals presented hepatic steatosis, which made it difficult to visualize hepatic fibrosis and this could underestimate the number of patients with some degree of fibrosis. It was found that individuals with periportal fibrosis were observed to present a larger portal vein diameter and it has been observed in other studies that patients with advanced periportal fibrosis in hepatosplenic forms presented a larger portal vein diameter [36]. However, patients in this present study did not present values larger than 1.20 centimeters, which is still within the normal reference range. The increased portal vein diameter is associated with the degree of periportal fibrosis and the disease severity [37]; however, it should not be interpreted as an isolated finding as it needs to be adjusted for body weight and may be a useful parameter for predicting severe complications, such as esophageal bleeding [38, 39].

When comparing infected individuals with no fibrosis and presenting fibrosis, we could not find an association related to spleen index and left and right liver lobes measurements. There has been reported association between advanced periportal fibrosis pattern and splenic enlargement in patients identified in hospital settings [40], where it is more likely to encounter patients with more severe, decompensated forms of the disease. However, in our study we only compared individuals identified in a population-based field survey who presented with compensated forms with schistosomiasis and found only one individual presenting pattern F fibrosis, which could explain our findings. Thus, this emphasizes the importance of conducting populational-based surveys and clinical evaluation in the context of schistosomiasis. Future evaluation should include individuals with more advanced forms to enrich clinical data on these populations, particularly in Pernambuco.

The frequency of periportal fibrosis among those who tested positive was higher in males, which is also reported in other studies published in the literature [41, 42]. Furthermore, individuals with periportal fibrosis presented with a higher mean and median ages and corroborate studies published in the literature [43, 44], which could be explained by the pathophysiology of the disease. Since hepatic fibrosis is a manifestation of chronic infection, younger individuals may not have been infected long enough for significant collagen deposition to develop hepatic fibrosis, which typically requires several years [45, 46]. Thus, it is assumed that the relationship between age and periportal fibrosis is linear [41]; however, in the area, individuals under 18 years of age were observed who already presented with type C fibrosis, which may be an indication of early exposure. This is supported by questionnaire data showing that most residents are involuntarily exposed to transmission sites during routine movement through streets that are frequently flooded during rainy periods. Furthermore, it shows the importance of conducting abdominal ultrasonography evaluation and early diagnosis through active case findings.

Among individuals with negative diagnostic tests, only 7.02% reported a history of schistosomiasis; however, 19 (33.33%) individuals presented periportal fibrosis on ultrasound examination. This discrepancy may be explained by the fact that the study area was previously targeted by a mass treatment program [16]. Consequently, individuals with prior infection and mild fibrotic lesions may have been treated while asymptomatic and therefore never formally diagnosed.

Some study limitations can be found in this study, such as, although the groups of registered non-participants and participants were similar on sex distribution, the participant group had a higher median age, as discussed before and should be considered when interpreting results. Also, due to infrastructure constraints, it was not possible to perform HPJ parasitological technique, molecular analysis by qPCR or ultrasound assessment for periportal fibrosis pattern on all individuals with negative samples by KK method. Nevertheless, the use of Kato-Katz is the recommended method by the WHO. Lastly, due to the cross-sectional nature of this study, it is not possible to infer causal relationships only by the results found here.

## Conclusion

The Novo Horizonte area presented a moderate positivity rate in a low endemicity municipality considered to have low endemicity. This population is under high frequency of exposure to disease transmission foci, considering the urban transmission profile recorded in the locality. These findings highlight the need for coproscopic surveys and strengthened active case detection in endemic municipalities, as well as the incorporation of more sensitive diagnosis methods in this context. Male individuals showed greater infection and periportal fibrosis frequency of periportal fibrosis. Furthermore, positive individuals presented larger portal vein diameter mean. In this context, more targeted studies are needed to better understand the dynamics of schistosomiasis transmission in this locality, as well as the health consequences for exposed and infected individuals.

## Data Availability

All data supporting the findings of this study are available within the paper.
